# The genetics of phenotypic plasticity. XIII. Interactions with developmental instability

**DOI:** 10.1002/ece3.1039

**Published:** 2014-03-18

**Authors:** Samuel M Scheiner

**Affiliations:** Division of Environmental Biology, National Science Foundation4201 Wilson Blvd., Arlington, Virginia, 22230

**Keywords:** Environmental heterogeneity, model, theory

## Abstract

In a heterogeneous environment, natural selection on a trait can lead to a variety of outcomes, including phenotypic plasticity and bet-hedging through developmental instability. These outcomes depend on the magnitude and pattern of that heterogeneity and the spatial and temporal distribution of individuals. However, we do not know if and how those two outcomes might interact with each other. I examined the joint evolution of plasticity and instability through the use of an individual-based simulation in which each could be genetically independent or pleiotropically linked. When plasticity and instability were determined by different loci, the only effect on the evolution of plasticity was the elimination of plasticity as a bet-hedging strategy. In contrast, the effects on the evolution of instability were more substantial. If conditions were such that the population was likely to evolve to the optimal reaction norm, then instability was disfavored. Instability was favored only when the lack of a reliable environmental cue disfavored plasticity. When plasticity and instability were determined by the same loci, instability acted as a strong limitation on the evolution of plasticity. Under some conditions, selection for instability resulted in maladaptive plasticity. Therefore, before testing any models of plasticity or instability evolution, or interpreting empirical patterns, it is important to know the ecological, life history, developmental, and genetic contexts of trait phenotypic plasticity and developmental instability.

## Introduction

In a heterogeneous environment, natural selection on a trait can lead to a variety of outcomes, depending on the magnitude and pattern of that heterogeneity and the spatial and temporal distribution and movement of individuals. Consider the following four possible outcomes: (1) multiple genotypes that express a different phenotype matching a particular selective optimum (genetic differentiation); (2) a single genotype that expresses a single intermediate phenotype (jack-of-all-trades); (3) a single genotype that expresses multiple phenotypes in response to environmental cues with each phenotype matching a different selective optimum (phenotypic plasticity); (4) a single genotype with offspring expressing randomly variable phenotypes some of which have high fitness (bet-hedging). (I refer to the process of producing randomly variable offspring as developmental instability and the selective outcome as bet-hedging.) These possibilities are not necessarily mutually exclusive, nor do they exhaust the named list of strategies that combine various aspects of each. For current purposes, however, they suffice to describe the range of possible evolutionary outcomes.

Of that range of possible outcomes, we would like to know which are more likely under particular circumstances. The first two (genetic differentiation and jack-of-all-trades) have been extensively explored, with models that go back to the origins of modern evolutionary theory (Hedrick et al. 1976). Models of the third and fourth (plasticity and bet-hedging) have been examined over the past three decades (Berrigan and Scheiner 2004; Starrfelt and Kokko 2012). This paper is the third in a set of three studying the intersection of phenotypic plasticity, developmental instability, environmental heterogeneity, and life-history strategy. The first paper (Scheiner 2013) examined phenotypic plasticity alone and explored the many ways that environmental variation could be created by the mode and pattern of spatial and temporal heterogeneity in combination with the mode and pattern of movement. The second paper (Scheiner 2014) did the same for developmental instability alone. Those papers set the stage for the current paper that explores the interaction of plasticity and instability. I strongly urge you to first read those papers and Scheiner and Holt (2012) to understand the context of this paper.

For the most part, phenotypic plasticity and developmental instability have been dealt with independently, both in models and empirical studies. In this paper, I explore a simulation model that allows for both outcomes, thus contrasting a deterministic genetic strategy—phenotypic plasticity—with a stochastic strategy—developmental instability. Two previous results stimulated this exploration, one theoretical and one empirical. Regarding theory, one of the unexpected results in our previous simulations (Scheiner and Holt 2012) was selection for hyperplasticity—a reaction norm much steeper than that expected by the environmental gradient. Hyperplasticity was favored when dispersal rates were high, individuals moved prior to selection, and temporal variation was high with a large negative autocorrelation. Under those conditions, there was great uncertainty and variability in the environment at the time of selection such that the optimal strategy was to produce highly variable offspring through plasticity. In effect, plasticity was acting as a form of het-hedging. This result raised the question about what conditions would select for the more classic form of bet-hedging, the production of randomly variable offspring, and how such a strategy might interact with or jointly evolve with plasticity.

The empirical observation was our demonstration that plasticity was positively genetically correlated with developmental instability in *Arabidopsis* (Tonsor et al. 2013). Although not the first such demonstration, it is the most detailed and involves the most traits. DeWitt et al. (1998) proposed that such a linkage could be a limitation on the evolution of plasticity. My model assumes that the same loci determine the amount of plasticity and the amount of instability, linkage by pleiotropy. Even in the absence of pleiotropy, the evolution of instability and plasticity might affect each other. Such interactions hitherto have been unexplored.

In this paper, I ask three questions. (1) Under what conditions is plasticity or instability more likely to evolve when both are possible outcomes? (2) How do the evolution of bet-hedging by developmental instability and phenotypic plasticity interact and trade-off with each other? (3) To what extent does developmental instability act as a limitation on the evolution of phenotypic plasticity when the two are pleiotropically linked?

### Patterns of environmental heterogeneity

The focus of my explorations has been on the myriad ways that environmental variation can combine with organismal biology to produce heterogeneity as perceived by an individual or experienced by a lineage. This myriad can be complex, resulting in a large parameter space to explore. The environment can vary in time and in space, alone or together. It can be uncorrelated or correlated in time and/or in space. Temporal variation can occur within the lifetime of an individual, or across generations. The biology of the organism creates two critical time periods, development (when the phenotype is determined) and selection, and individuals can move before or after selection occurs. That movement can be local (to adjacent demes only), or can be global (to any deme).

**Figure 1:**
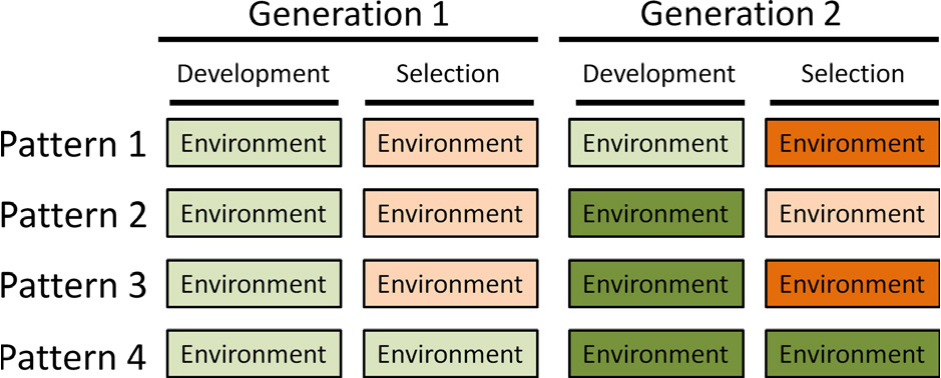
Patterns temporal environmental variation used in various simulations. (1) The environment of development is fixed, and the environment of selection varies among generations. (2) The environment of development varies among generations, and the environment of selection is fixed. (3) Both the environment of development and the environment of selection vary among generations independently of each other. (4) The environment of development and the environment of selection are identical within a generation and vary among generations. For all of these patterns, the among-generation correlation could vary from positive to negative. Movement could occur once, either after development and before selection (*move first*) or after selection (*select first*). Across space, the temporal variation could occur independently in each deme, or could be synchronized across all demes.

Let us deconstruct that complexity. With respect to the evolution of phenotypic plasticity, all variation must be considered relative to when the phenotype is determined, movement occurs, and selection happens (Fig. 1). In my model, phenotypic determination is treated as a developmental stage such that the phenotype is fixed at some point in the life history. Assuming that an organism's phenotype is fixed following development, we can define two scales of temporal variation: (1) Among-generation change in the environment can happen at the time of development or at the time of selection, or both. (2) Within-generation change can happen between the time of development and the time of selection. Similarly, if an organism moves once during its life and selection occurs at a single instance, we can define two life-history patterns: selection then movement, and movement then selection. These two patterns, along with the presence or absence of spatial heterogeneity, define the types of environmental heterogeneity that are relevant to the evolution of phenotypic plasticity. The types relevant to the evolution of developmental instability are a subset of those because the environment plays no role in development; thus, the only environmental change that matters is variation at the time of selection.

I consider three combinations of environmental heterogeneity: (1) temporal variation alone; (2) spatial variation alone; and (3) both temporal and spatial variation. When temporal variation occurs alone, it happens in a single deme once per generation before development and can be independent from one generation to the next or it can be autocorrelated. When spatial variation occurs, it is among demes along a linear gradient producing a spatial autocorrelation. That spatial heterogeneity can be overlaid by temporal variation within demes such that the spatial pattern is a central tendency, that is, present but varying in magnitude and pattern. I consider four broad patterns that combine spatial and temporal variation (Fig. 1).

In the first pattern, the environment of development is fixed among generations, while the environment of selection varies among generations. In the second pattern, the environment varies among generations at the time of development, while the environment of selection is fixed. In the third pattern, the environment changes both at the time of development and at time of selection and those changes are not correlated. In the fourth pattern, environmental change at the time of development carries over to the environment at selection. For each of those four patterns, the temporal variation could be synchronized among demes or occur independently in each deme.

Finally, I consider two different movement patterns: stepping-stone migration and island migration. For stepping-stone migration, movement occurs among nearby demes with the probability of movement decreasing with distance. For island migration, movement occurs among all demes with equal probability, although more complex movement rules are possible. See Scheiner (2013) for a detailed discussion of these patterns and possible ecological scenarios that they represent.

### Phenotypic plasticity and developmental instability alone

My previous explorations of the evolution of plasticity and instability when each was evolving alone found the following. For phenotypic plasticity, among-generation temporal heterogeneity favored plasticity, while within-generation heterogeneity, especially at the time of development, resulted in cue unreliability disfavoring plasticity. In general, spatial variation more strongly favored plasticity than temporal variation, and island migration more strongly favored plasticity than stepping-stone migration.

The pattern of evolutionary response can be quite complex when individuals move in space after development but before selection. Negative correlations among environments between the time of development and selection resulted in seemingly maladaptive reaction norms. When movement occurred before selection, the dispersal rate was high, and temporal variation was large, selection favored hyperplasticity—reaction norms much greater than the fitness optimum. This hyperplasticity was favored because it increases the phenotypic range of a lineage, acting as a form of bet-hedging.

For developmental instability, both temporal and spatial heterogeneity selected for instability. For spatial heterogeneity, the response to selection depended on the life-history strategy and the form and pattern of dispersal with the greatest response for island migration when selection occurred before dispersal. Combining spatial and temporal heterogeneity resulted in substantially more instability than either alone. Positive correlations among generations reduced that response, while synchronizing that temporal change among demes more strongly favored instability but eliminated the synergy between temporal and spatial heterogeneity.

Thus, patterns of environmental heterogeneity can be complex and can interact in unforeseen ways. Temporal and spatial variation do not combine additively but depend on the life-history pattern. For both plasticity and instability, I found higher-order interactions between life-history patterns, dispersal rates, dispersal patterns, and environmental heterogeneity.

### Model structure

My model is an individual-based simulation implemented in Fortran 77 (the computer code is available from Dryad, doi:10.5061/dryad.9 bp88). A summary of parameters is given in Table 1.

**Table 1:** Summary of the model parameters.

Fixed parameters
Number of nonplastic, plastic, and developmental instability loci = 5 each
Steepness of the gradient (change in optimum in adjacent demes) = 0.4 units
Strength of selection within demes (σ) = 2 units
Mutation rate = 10%/allele/generation
Mutational effect (standard deviation) = 0.1 units
Number of generations = 10,000
Parameters explored
Length of the environmental gradient: 1 or 50 demes
Population size: 1000 or 100 individuals/deme
Life-history pattern: selection before dispersal versus dispersal before selection
Timing of environmental change: at the time of development versus selection
Dispersal pattern: stepping-stone or island
Dispersal rate (5–84%)
Magnitude of environmental change (0–50% of the length of the spatial gradient)
Correlation of environmental change among generations (−0.75–0.75)
Strength of the pleiotropic effect of plasticity on developmental instability (0–4)

The genotype of an individual consisted of up to three types of loci: (1) genes with deterministic expression that was independent of the environment (nonplastic loci); (2) genes with deterministic expression that was dependent of the environment (plastic loci); and (3) genes that allowed for random deviation from the deterministic phenotype (instability loci). In some versions of the model, plasticity and developmental instability were determined by the same loci. This combination of loci types allows for all four possible evolutionary outcomes: genetic differentiation, jack-of-all-trades, plasticity, and bet-hedging. For example, adaptation by genetic differentiation would occur when the allelic values of the plastic and instability loci go to zero (i.e., are not expressed). Conversely, adaptation by phenotypic plasticity would occur when the allelic values of the nonplastic determinstic loci and the instability loci go to zero. Intermediate outcomes were also possible in which individuals express the optimal phenotype in a particular environment through nonzero values of any combination of the nonplastic, plastic, and instability loci.

For temporal-only heterogeneity, the metapopulation consisted of a single deme. For spatial heterogeneity, the metapopulation consisted of a linear array of 50 demes. An environmental gradient was created by varying the optimal value of a single trait (phenotype) in a linear fashion along the array from −9.8 to +9.8 arbitrary units at the ends of the gradient, that is, the optimal phenotype in adjacent demes differed by 0.4 units. An individual's phenotype (trait value) was determined by 15 diploid loci: five nonplastic loci, five plastic loci, and five instability loci. The deterministic loci contributed additively to the trait. Allelic values at the plastic loci were multiplied by an environment-dependent quantity before being summed. The effect of the environment (*E*_*i*_ for deme *i*) on the phenotypic contribution of each unit plastic allelic value varied in a linear fashion, with a slope of 0.04 units [*E*_*i*_ = 0.04(*i* − 25.5)]. The phenotype of each individual was determined at the time of development as

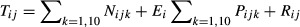
1

where *T*_*ij*_ is the phenotype of the *j*th individual that develops in the *i*th deme, *N*_*ijk*_ is the allelic value of the *k*th nonplastic allele of that individual, *P*_*ijk*_ is the allelic value of the *k*th plastic allele, and *R*_*ij*_ is the developmental instability of that individual. For a given genotype, Σ*N*_*ijk*_ can also be thought of as the intercept of its reaction norm, or the phenotype of the individual in the absence of plasticity, and the slope of [*E*_*i*_Σ*P*_*ijk*_] calculated across demes can be thought of as the slope of its reaction norm. For the parameters used here, the optimal reaction norm had a slope of 10.

For developmental instability that was not due to the pleiotropic effects of plasticity, *R*_*ij*_ was determined by a Gaussian normal deviate with a standard deviation equal to ∑_*k* = 1,10_*D*_*ijk*_, where *D*_*ijk*_ is the allelic value of the *k*th instability allele. For instability that was due to pleiotropy, *R*_*ij*_ = *S*|∑ _*k*=1,10_*P*_*ijk*_|, where *S* is a Gaussian normal deviate with a standard deviation equal to the scaled effect of plasticity multiplied by the absolute value of the genotypic value of plasticity. Individuals with equal amounts of plasticity but opposite slopes have the same effect on developmental instability.

Life-history events occurred in one of the following two sequences: (1) birth, followed by development (i.e., the phase in the life cycle when the phenotype is determined), then dispersal, selection, and reproduction (denoted as “move first”); or alternatively; (2) birth, development, selection, dispersal, and then reproduction (denote as “select first”). Selection was based on survival with the probability of surviving being a Gaussian function of the difference between an individual's phenotype and the locally optimal phenotype. Fitness (the probability of surviving) was determined as follows:

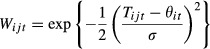
2where *W*_*ijt*_ is the fitness of the *j*th individual in the *i*th deme in generation *t*,*T*_*ijt*_ is the phenotype of that individual, *θ*_*it*_ is the optimal phenotype in that deme, and σ is the strength of selection (selection weakens as σ increases). For all simulations, σ = 2; the length of the spatial gradient across all demes was approximately five times the width of the within-deme selection function.

Temporal variation occurred at one or both of two life-history stages: at the time of development or at the time of selection. This variation could occur once per generation at one of the two stages with the environment remaining fixed at the other stage or could occur at both stages. If the variation occurred at both stages, those changes could be independent or they could carry through both stages. Finally, the changes could be independent among demes, or be synchronized among demes. In the latter case, the optimal phenotype in all demes changed by the same magnitude and direction. That variation could be uncorrelated from one generation to the next or be correlated among generations. If variation was independent among demes, each deme had its own pattern of temporal autocorrelation.

Temporal autocorrelation was simulated using the recursion:

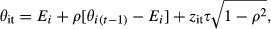
where *θ*_*it*_ is the environment at either development or selection in the *i*th deme in generation *t*,*E*_*i*_ is the mean or fixed environment in the *i*th deme (a linear function of *i*), τ is the standard deviation of environmental variation, ρ is the temporal autocorrelation coefficient, and *z*_*it*_ is a sequence of independent zero-mean, unit-variance Gaussian random deviates. For simulations without temporal variation, *τ* = 0, and for uncorrelated temporal variation, *ρ* = 0. The standard deviation of environmental noise (*τ*) is shown as a percentage of the difference in the optima at the two ends of the gradient. The autocorrelation (*ρ*) varied from −75% to 75%.

Dispersal occurred in one of the two patterns: stepping-stone or island. For the stepping-stone migration pattern, the dispersal probability and the distance moved were determined using a zero-mean Gaussian random number, so that the probability of moving and the average distance moved were correlated (see Fig. 1 of Scheiner and Holt 2012). Increasing the dispersal probability was performed by increasing the variance of the Gaussian so that both more individuals were likely to move, and they were likely to move farther. Individuals that would otherwise disperse beyond the end of the gradient moved to the terminal demes. For the island migration pattern, each individual had a fixed probability of moving. If it moved, it had an equal probability of moving to any of the other demes. For both patterns, dispersal per se had no cost; survival during dispersal was 100%. The dispersal probabilities were the same for the two life-history strategies, *move first* and *select first*; however, the absolute number of individuals dispersing was fewer for the *select first* life-history strategy because of reductions in deme sizes due to selection.

Reproduction was accomplished by assembling pairs of individuals within a deme at random with replacement (allowing for self-fertilization), with each pair producing 1 offspring, then repeating until the carrying capacity of that deme was reached. This procedure assumes soft selection, in that local population size was determined independently of the outcome of selection, and results in individuals competing for offspring. The model assumes that the spatial scale of reproduction and mating matches that of density dependence and the grain of the selective environment.

Each simulation was initialized with 100 individuals being born in each deme, or 1000 individuals for simulations with just temporal variation in a single deme. Those deme and total population sizes (5000 individuals (50 × 100) and 1000 individuals, respectively) were chosen to minimize genetic drift and the effects of population size on among-individual fitness variance while making the simulations computationally tractable. For each individual in the initial generation, allelic values for both plastic and nonplastic loci were chosen independently from the values −2, −1, 0, 1 and 2, with each value being equally likely. (Even though initial values are discrete, due to mutation allelic values are continuous variables after the initial generation; see below.) When new offspring were generated, each allele mutated with a probability of 10%. [Lower mutation rates mainly changed the time-scale over which evolution occurs, rather than the eventual outcome (Scheiner and Holt 2012).] When a mutation occurred, the allelic value was changed by adding a Gaussian deviate (mean of zero and a standard deviation of 0.1 units) to the previous allelic value (i.e., this is an infinite-alleles model). For the developmental instability loci, all alleles began with a value of zero. The probability of mutation and the standard deviation of the Gaussian deviate were the same as for the other loci, but only positive allelic values were retained, negative values were set to zero.

All simulations were run for 10,000 generations to ensure that the equilibrium point (the point after which all calculated quantities showed no further directional trend) was reached. Each parameter combination was replicated 20 times, and the results shown are the means of those replicates. Coefficients of variation of reported parameters were generally low (1–5%). If the metapopulation went extinct, additional realizations were run until 20 successful replications were achieved; for some parameter combinations, the extinction probability was 100% (i.e., no successful replications in 60 runs). Reported outcomes were averaged over successful replications only.

The reaction norm describes how the phenotypic expression of a given genotype varies among environments. The plasticity of a linear reaction norm is best described by its slope. In this model, the slope of the reaction norm is the product of the slope of *E*_*i*_ across demes and the sum of the values of the plasticity alleles (i.e., the right-hand sum in eq. 1). For these simulations, as the slope of *E*_*i*_ was constant, and the final outcome was measured as the average across all demes of the sum of the values of the plasticity alleles for each individual. That is, 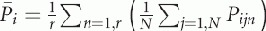, where 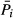 is the mean plasticity of the *i*th deme over all *r* runs, *N* = 100 is the number of individuals per deme, and *P*_*ijn*_ is the sum of the values of the plasticity alleles of the *j*th individual developing in the *i*th deme in the *n*th run. The overall mean plasticity 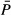 is the average of 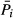 across demes and is given by 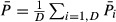, where *D* is the number of demes. (The order of averaging, over runs within demes first or over demes within runs first, does not affect the final average, because the number of demes is the same for all runs.) The average plasticity was standardized as relative plasticity to the optimal reaction norm (the slope of *E*_*i*_) so that a pure plasticity outcome would have a value of 1 and a pure differentiation outcome would have a value of 0. Values outside this range were possible; that is, it was possible to achieve a reaction norm with a slope steeper than the optimal value (>1) or in a direction opposite from the optimal value (<0).

## Results

### Temporal heterogeneity only

**Figure 2:**
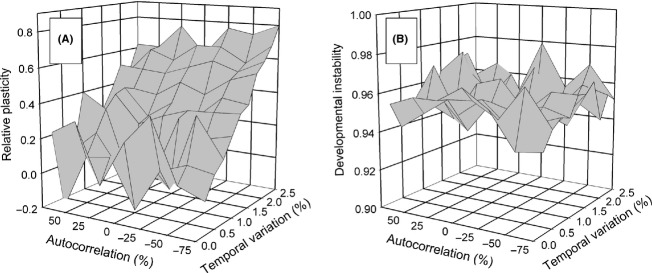
The effect of temporal variation alone on selection for phenotypic plasticity (mean *P*_*ij*_) and developmental instability (mean *R*_*ij*_) when change occurs once per generation at the time of development and carries through to selection (pattern 4). Temporal variation is scaled relative to the strength of selection (τ/σ). (A) The effect on plasticity; a relative plasticity value of 1.0 indicates a pure plasticity outcome. (B) The effect on developmental instability.

When there was only temporal heterogeneity (i.e., a single deme) and the environment changed once per generation before development (pattern 4), phenotypic plasticity (mean *P*_*ij*_) was selected for in the same way as when only plasticity loci were present [Fig. 2A, compare with Fig. 1A of Scheiner (2013)]. In contrast, selection for developmental instability (mean *R*_*ij*_) was completely suppressed [Fig. 2B, compare with Fig. 2 of Scheiner (2014)]. Developmental instability was at its selection-mutation balance, approximately 0.93 for the parameter values used here. When the environmental cue was not reliable because change occurred both at the time of development and the time of selection (pattern 3), there was selection against plasticity, just as when only plasticity loci were present [Fig. S1A, compare with Fig. 1B of Scheiner (2013)]. However, developmental instability was also selected against (Fig. S1B).

### Spatial heterogeneity only

**Figure 3:**
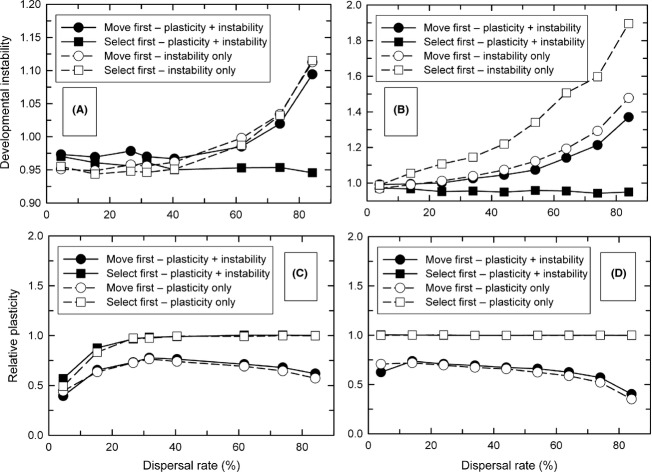
The effect of spatial variation alone on selection for developmental instability (mean *R*_*ij*_) and phenotypic plasticity (mean *P*_*ij*_) either independently or together. (A) and (C) Stepping-stone migration; (B) and (D) island migration.

When there was only spatial heterogeneity, the response to selection on developmental instability (mean *R*_*ij*_) depended on the life-history strategy (Fig. 3). For the *select first* life-history strategy, when plasticity loci were also present selection for instability disappeared. For the *move first* life-history strategy, the presence of plasticity loci diminished instability only slightly. These effects occurred for both dispersal patterns. In contrast, selection for plasticity (mean *P*_*ij*_) was unaffected by the presence of instability for either life-history strategy or dispersal pattern. In general, when the metapopulation achieved the optimal reaction norm, instability was disfavored. Otherwise, selection resulted in a mixed outcome of plasticity and bet-hedging through instability.

### Spatial and temporal heterogeneity—effects on plasticity

**Figure 4:**
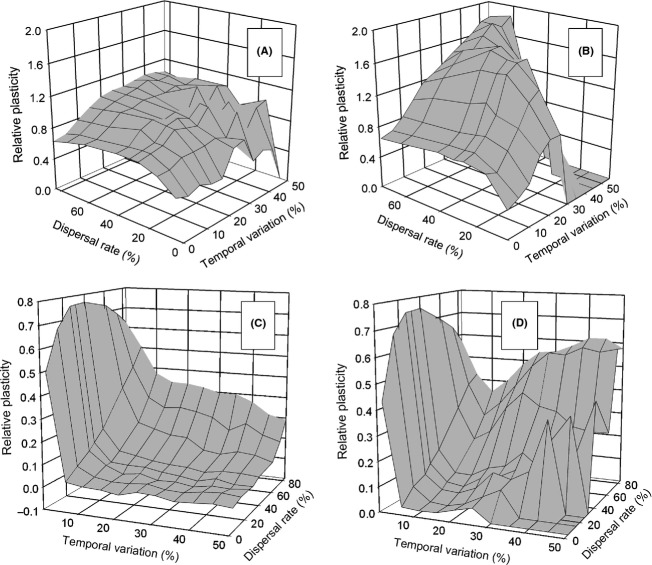
The interaction of dispersal rate and temporal variation of the local phenotypic optima on the evolution of phenotypic plasticity (mean *P*_*ij*_) when dispersal occurs before selection (*move first*). Temporal variation is scaled as a percentage of the length of the environmental gradient and was independent among demes. Dispersal was by the stepping-stone migration pattern. (A) and (B) The environment changed once per generation at the time of selection (pattern 1). (C) and (D) The environment changed at the time of development and again at the time of selection (pattern 3). (A) and (C) With developmental instability; (B) and (D) with plasticity alone. For the clarity of viewing, the axes are reversed between (A) and (B) versus (C) and (D). The data shown in (B) and (D) were first published in the study described by Scheiner (2013) and are presented here for comparative purposes.

When there was both temporal and spatial heterogeneity, the result depended on the timing and pattern of that temporal variation. Developmental instability affected the evolution of phenotypic plasticity (mean *P*_*ij*_), when the environment of selection varied (Fig. 1, patterns 1 and 3), only for the *move first* life-history strategy, and only when temporal variation and dispersal rates were high. For pattern 1, the addition of instability completely eliminated selection for hyperplasticity, instead resulting in the optimal reaction norm (Fig. 4A and B). For pattern 3, the addition of instability resulted in little or no plasticity when temporal variation was high (Fig. 4C and D).

In contrast, for the *select first* life-history strategy, these environmental patterns resulted in responses identical to those when instability was absent [compare Fig. S2A and B with Fig. 3A and Fig. 4A, respectively, of Scheiner (2013)]. For the other environmental patterns—variation at the time of development only (pattern 2) and variation at the time of development that carries over to selection (pattern 4)—and both life-history strategies, the evolutionary responses were identical to those when instability was absent (results not shown).

### Spatial and temporal heterogeneity—effects on instability

**Figure 5:**
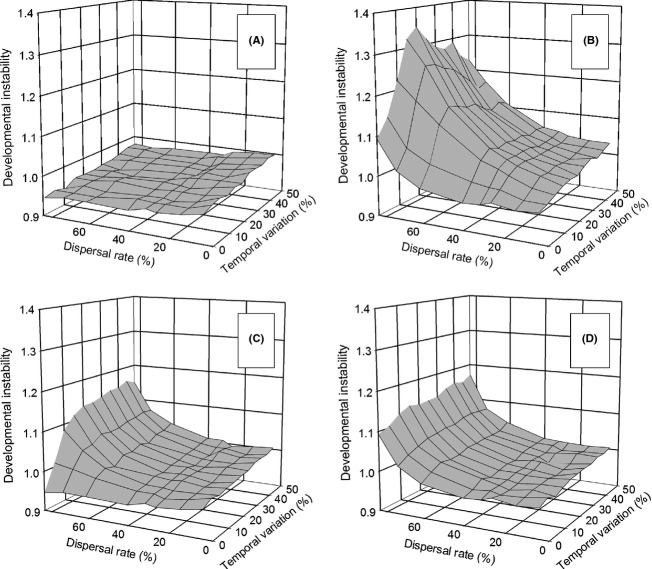
The propensity for developmental instability (mean *R*_*ij*_) to be favored by selection when plasticity is present and there is both temporal and spatial environmental heterogeneity. Temporal variation is scaled as a percentage of the length of the environmental gradient and was independent among demes. Dispersal was by the stepping-stone migration pattern. (A) and (B) Environmental change occurred once per generation at the time of development and carried over to selection (pattern 4). (C) and (D) Environmental change occurred once per generation at the time of development only (pattern 2). (A) and (C) Selection before dispersal (*select first*); (B) and (D) dispersal before selection (*move first*).

Phenotypic plasticity affected the evolution of developmental instability only when the environment varied at the time of development. When the environment changed at the time of development and that change carried through to selection (Fig. 1, pattern 4), there was little to no selection for instability (Fig. 5A and B) as compared to the situation when plasticity was absent (see Fig. 4C and D of Scheiner 2014). As with spatial heterogeneity alone, for the *select first* life-history strategy, selection for instability was completely suppressed. For the *move first* life-history strategy, there was maximal selective response at high dispersal rates and intermediate temporal variation. The reason for the weak selection on developmental instability was that the metapopulation achieved the optimal reaction norm under nearly all conditions (Fig. S3).

When the environment changed at the time of development only (pattern 2), the selective optima do not vary in time, just in space. As expected, dispersal rate affected selection on instability in a fashion similar to spatial variation alone (Fig. 5C and D). In the previous scenarios, change was independent among the demes. If those changes were synchronized among the demes, selection on instability was affected only when the environment changed at the time of development and that change carried through to selection (pattern 4) and only for the *move first* life-history strategy. In this instance, the response was similar to that for pattern 2 (results not shown).

For the other environmental patterns—variation at the time of selection only (pattern 1) and variation both at the time of development and at the time of selection (pattern 3)—and both life-history strategies, the effects of dispersal rate and the amount of temporal variation were identical to those when plasticity was absent (results not shown). For these last two patterns, the evolutionary outcome was a mixture of both plasticity and bet-hedging through instability.

### Pleiotropic developmental instability

**Figure 6:**
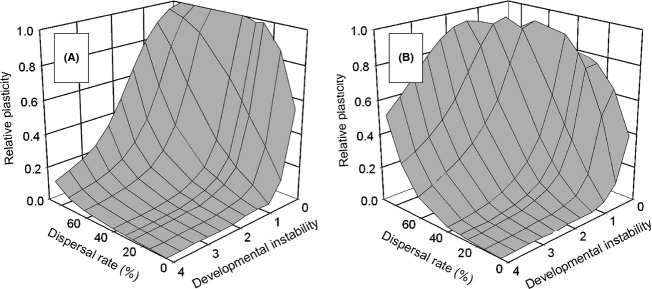
The effect of pleiotropic developmental instability on the evolution of phenotypic plasticity (mean *P*_*ij*_), when there is only spatial heterogeneity. Developmental instability (*S*) is scaled to the standard deviation of the random component of phenotypic variation if the metapopulation equals the optimal reaction norm. Dispersal was by the stepping-stone migration pattern. (A) Selection before dispersal (*select first*); (B) dispersal before selection (*move first*).

If developmental instability is a pleiotropic outcome of phenotypic plasticity, it acts as a selective limitation on plasticity. With temporal variation only, the population evolved to be nonplastic, a jack-of-all-trades outcome (results not shown). Similarly, with spatial variation only, plasticity declined rapidly with increasing instability for the *select first* life-history strategy (Fig. 6), resulting in genetic differentiation when dispersal rates were low and a jack-of-all-trades when dispersal rates were high. The decline in plasticity was ameliorated somewhat at very high dispersal rates, even more so for the *move first* life-history strategy. The latter conditions favored genetic differentiation over a jack-of-all-trades. Because the actual developmental instability experienced by an individual depends on the amount of plasticity, in these graphs developmental instability is scaled so that the standard deviation of the random component of phenotypic variation (*S*) equals that of a metapopulation with the optimal reaction norm. That is, when relative plasticity equals 1, the amount of instability (*S*) is equivalent to that of Fig. 5.

**Figure 7:**
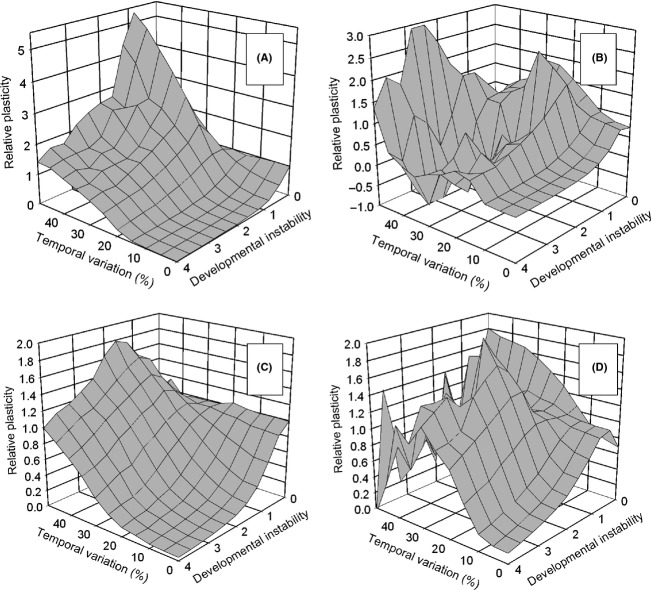
The effect of pleiotropic developmental instability on the evolution of phenotypic plasticity (mean *P*_*ij*_) when there is both temporal and spatial heterogeneity. Dispersal was by the stepping-stone migration pattern. Developmental instability (*S*) is scaled to the standard deviation of the random component of phenotypic variation if the metapopulation equals the optimal reaction norm. The environment changed once per generation at the time of selection only (pattern 1) and was independent among demes. (A) and (C) Selection before dispersal (*select first*); (B) and (D) dispersal before selection (*move first*). (A) and (B) Dispersal rate = 32%; (C) and (D) dispersal rate = 64%.

The addition of temporal variation at the time of selection altered this pattern. For the *select first* life-history strategy, at high temporal variation and low instability, there was selection for hyperplasticity. This effect was greatest at moderate dispersal rates (32%, Fig. 7A) but also occurred at high dispersal rates (64%, Fig. 7C) with the peak shifting to somewhat higher instability rates. This result contrasts with that when developmental instability was not genetically linked to plasticity, which selected for the optimal reaction norm (Fig. S3A). At very high amounts of pleiotropic instability, there was selection for intermediate to optimal reaction norms.

For the *move first* life-history pattern, the results were more complex (Fig. 7B and D). At levels of temporal variation of 20–25% and above, the results were idiosyncratic. For moderate dispersal rates and high levels of temporal variation (Fig. 7B), plasticity ranged from 3 (extreme hyperplasticity) to −1 (maladaptive plasticity), with no apparent pattern. As expected, there was an accompanying rise in the variation among runs as indicated by a 5–10 fold increase in the among-run coefficient of variation in plasticity (results not shown). This increase likely indicates that the metapopulation was not reaching equilibrium, even after 10,000 generations, possibly because of conflicting selection for very high levels of instability and intermediate levels of plasticity.

**Figure 8:**
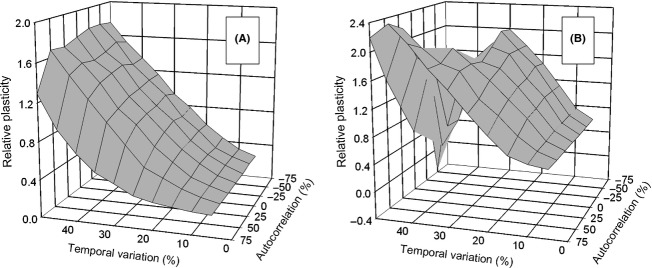
The interaction of temporal variation and among-generation correlation on the effect of pleiotropic developmental instability on the evolution of phenotypic plasticity (mean *P*_*ij*_). Dispersal was by the stepping-stone migration pattern. The environment changed once per generation at the time of selection only (pattern 1) and was independent among demes (dispersal rate = 64%, developmental instability (*S*) = 1.6). (A) Selection before dispersal (*select first*); (B) dispersal before selection (*move first*).

I also examined the effect of an among-generation autocorrelation in the temporal variation under conditions where the effect of instability was moderate and the dispersal rate was high (Fig. 8). For the *select first* life-history strategy, there was little effect of autocorrelation except when it was strongly positive. For the *move first* life-history pattern, at high temporal variation, positive autocorrelation selected for hyperplasticity, while negative autocorrelation selected for no plasticity. These results contrast with that when there was no instability where the greatest hyperplasticity was associated with negative autocorrelation (see Fig. 2C and D of Scheiner 2013).

## Discussion

When phenotypic plasticity and developmental instability are allowed to jointly evolve, the general effect is either for one to have no effect on the other, or for one to suppress selection for the other. Overall, the result is less plasticity or instability than when either is evolving in isolation of the other. However, those effects depend on the pattern of environmental heterogeneity, the life-history strategy, with some combinations resulting in a mixture of both plasticity and instability (e.g., Fig. 3). Genetic linkage between plasticity and instability further complicates these outcomes.

### When genetically independent

When plasticity and instability are determined by different loci, the only effect on the evolution of plasticity is the elimination of plasticity as a bet-hedging strategy (Fig. 4). This bet-hedging effect occurs when the environment varies at the time of selection independently of the environment at the time of development (patterns 1 and 3) and individuals move prior to selection (*move first*). This result confirms our previous conclusion that hyperplasticity can be a form of bet-hedging (Scheiner and Holt 2012). However, plasticity is less effective as a form of bet-hedging because it creates variation only among related individuals that develop in different environments, not among siblings in the same environment.

When the environment of development provides a reliable cue to the environment of selection (e.g., the *select first* life-history strategy), phenotypic plasticity is favored [theory of the evolution of phenotypic plasticity proposition 4, see Appendix of Scheiner (2013)]. Random variation in the phenotype of individuals has no effect on that cue reliability because reliability is a function of the central tendency of the environment. This result confirms the accuracy of our previous models (Scheiner et al. 2012; Scheiner and Holt 2012; Scheiner 2013) that did not include a random component to the phenotype.

In contrast, the effects of plasticity on the evolution of instability were more substantial. Temporal variation alone disfavored instability whether or not plasticity was favored; spatial variation alone or in combination with temporal variation was needed for instability to be favored. This result differs from those for instability alone (Scheiner 2014) and contrasts strongly with other models of the evolution of bet-hedging that predict that temporal variation will favor instability more than spatial variation (Starrfelt and Kokko 2012). Even when there is spatial variation, if conditions are such that the population is likely to evolve to the optimal reaction norm, then instability is disfavored (e.g., for the *select first* life-history strategy). When plasticity is less favored (e.g., for the *move first* life-history strategy) or not favored at all (e.g., pattern 2 when temporal variation is high), then instability will be favored. These results makes intuitive sense as there is no need for bet-hedging when the population evinces the optimal reaction norm and has maximal fitness. Under those circumstances, any random phenotypic variation tends to move individuals away from the optimal phenotype.

### When genetically linked

When plasticity and instability are determined by the same loci, instability can act as a strong limitation on the evolution of plasticity (theory of the evolution of phenotypic plasticity proposition 6). Even small affects of plasticity on instability result in selection against plasticity (Fig. 6). This linkage is one explanation for why plasticity is less ubiquitous than we might expect (K. Palacio-López, B. Beckage, S. Scheiner and J. Molofsky unpubl. ms.).

When temporal variation is combined with spatial variation, there appears to be selection for maladaptive plasticity. However, I interpret these results as selection for instability, rather than selection for plasticity. When selection occurs before dispersal (*select first*), there should never be selection for hyperplasticity, yet that was the observed response (Figs. 7A,C and 8A). One reason that instability was favored under these conditions is that it reduces the probability of the metapopulation going extinct (Scheiner 2014), another bet-hedging effect. For the *move first* life-history strategy, the evolutionary response is best characterized as “complex” (Figs. 7B, D and 8B), possibly because of conflicting selection for instability and plasticity, and will require an analytic model to tease apart what is selection for plasticity and what is selection for instability.

## Conclusions

In answer to the questions posed at the beginning of this paper: (1) Plasticity is more likely to evolve than instability any time that there is a reliable environmental cue. (2) Bet-hedging through plasticity is eliminated by the presence of instability and, similarly selection for instability is eliminated when phenotypic plasticity can maintain the optimal phenotype. The two types of evolutionary responses to heterogeneous environments are negatively synergistic. (3) When the two are pleiotropically linked, developmental instability acts as a limitation on the evolution of phenotypic plasticity. This is the first formal proof of that conjectured limitation put forth by DeWitt et al. (1998).

My model predicts that in empirical systems we should find a negative correlation between phenotypic plasticity and developmental instability when the two are genetically independent. Whether that negative correlation will be found among traits within a single population, or across populations within a single trait, depends on the genetic architecture of plasticity and instability. For example, it will depend on whether the plasticity of one trait is pleiotropically linked to the plasticity of other traits. While there is now a substantial literature concerning the genetic bases for plasticity (DeWitt and Scheiner 2004), there is little information regarding the genetics of instability. We know that developmental instability can have a genetic basis (Scheiner et al. 1991; Ros et al. 2004; Ibáñez-Escriche et al. 2008; Shen et al. 2012). However, evidence of a genetic linkage between plasticity and instability is equivocal with a few studies finding a positive relationship for some traits (Waddington and Robertson 1966; Perkins and Jinks 1973; Garcia-Vázquez and Rubio 1988; Scheiner et al. 1991; Tonsor et al. 2013), but others failing to find any relationship (Waddington 1960; Kindred 1965; Scharloo et al. 1972; Bagchi and Iyama 1983; Santiago et al. 1989). All of those studies used a quantitative genetics approach, strongly suggesting that molecular genetic information will prove enlightening.

That predicted negative correlation is predicated on particular patterns of environmental heterogeneity and life-history strategies. Testing that prediction using correlations among populations requires that they all experience the same temporal and spatial patterns of environmental heterogeneity. Testing the prediction using correlations among traits, within a single population has the advantage of ensuring that the ecology of the organism is the same for all traits. However, even that simplicity is tricky if the timing of developmental decisions differs among traits. In that case, an environment that is fixed at the time of development for one trait may well be variable at the time of development of another trait.

My model is the first to consider the joint evolution of phenotypic plasticity and developmental instability. The results have implications for testing previous models of their independent evolution. For models of plasticity evolution, what matters most is whether plasticity and instability are genetically linked. If not, then it is only a small part of parameter space where trait instability will have an effect. For the models of instability evolution, what matters most is whether phenotypic plasticity allows trait values to match the phenotypic optimum under most conditions, thus eliminating selection for instability, although genetic linkage is also important. I therefore expand my previous conclusions (Scheiner 2013, 2014) about the importance of knowing the ecological and life-history context of trait plasticity and instability. It is also necessary to know the developmental and genetic context of any trait. Measurements of plasticity or instability divorced of such context (e.g., measured under limited growth chamber or greenhouse conditions), may be very misleading tests of such models or characterizations of patterns in natural populations.

## Supporting Information

**Figure S1.** The effect of temporal variation alone on selection for phenotypic plasticity (mean *P*_ij_) and developmental instability (mean *R*_ij_) when change occurs both at the time of development and the time of selection (pattern 3). **Figure S2.** The propensity for phenotypic plasticity (mean *P*_ij_) to be favored by selection when developmental instability is present and there is both temporal and spatial environmental heterogeneity. **Figure S3**. The interaction of dispersal rate and temporal variation of the local phenotypic optima on the evolution of phenotypic plasticity (mean *P*_ij_) when selection occurs before dispersal (*select first*).
