# Supplementary material for: The genetics of phenotypic plasticity. XIII. Interactions with developmental instability
Source: Ecol Evol. 2014 Mar 18;4(8):1347–60. doi: 10.1002/ece3.1039 (PMC4020694; doi:10.1002/ece3.1039)
Supplement: Supplementary file 1 — Figure S1. The effect of temporal variation alone on selection for phenotypic plasticity (mean Pij) and developmental instability (mean Rij) when change occurs both at the time of development and the time of selection (pattern 3). Figure S2. The propensity for phenotypic plasticity (mean Pij) to be favored by selection when developmental instability is present and there is both temporal and spatial environmental heterogeneity. Figure S3. The interaction of dispersal rate and temporal variation of the local phenotypic optima on the evolution of phenotypic plasticity (mean Pij) when selection occurs before dispersal (select first). [file ece30004-1347-sd1.pdf]

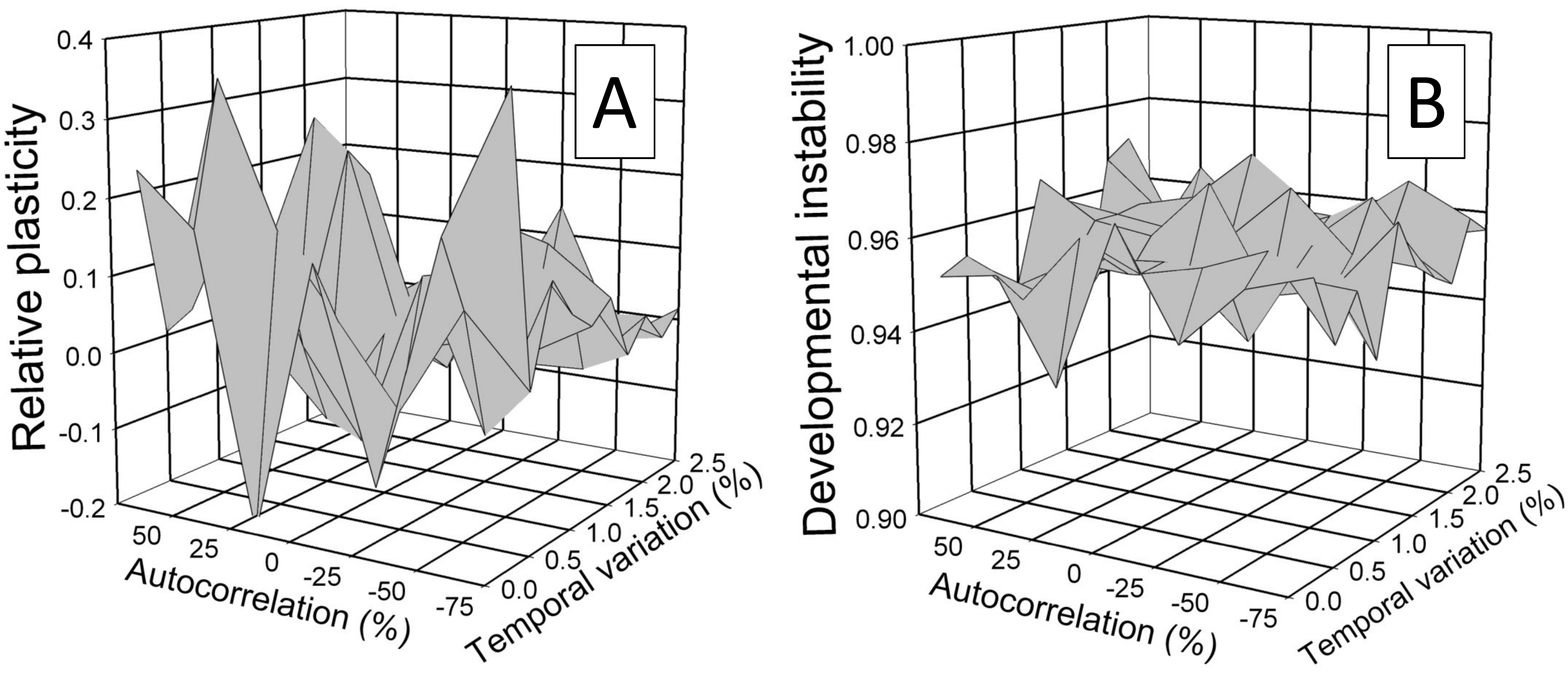

Figure S1. The effect of temporal variation alone on selection for phenotypic plasticity (mean P_{ij}) and developmental instability (mean R_{ij}) when change occurs both at the time of development and the time of selection (pattern 3). Temporal variation is scaled relative to the strength of selection (τ/σ). (A) The effect on plasticity; a relative plasticity value of 1.0 indicates a pure plasticity outcome. (B) The effect on developmental instability.

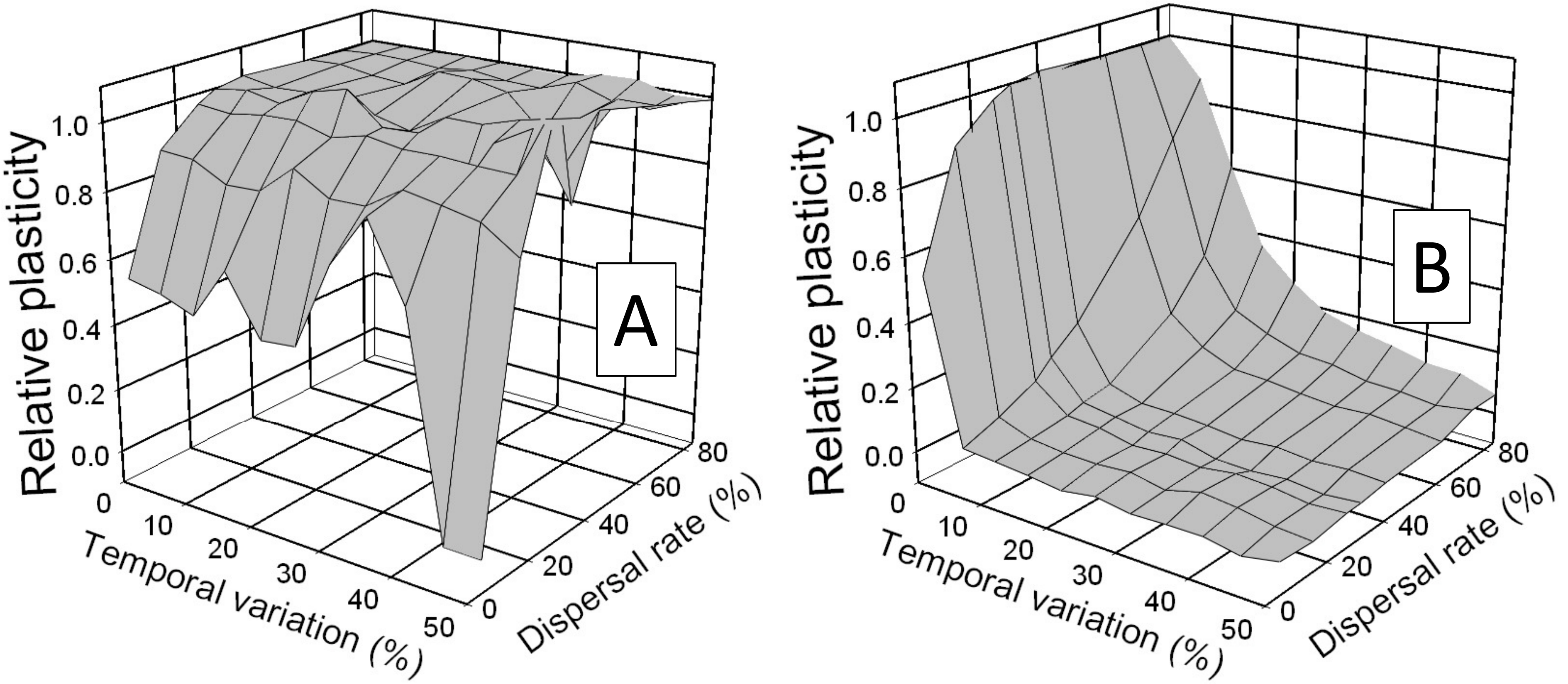

Figure S2. The propensity for phenotypic plasticity (mean P_{ij}) to be favored by selection when developmental instability is present and there is both temporal and spatial environmental heterogeneity. Environmental change occurred once per generation before development and carried over to selection (pattern 4) and was independent among demes. Dispersal was by the stepping-stone migration pattern. (A) Selection before dispersal (*select first*); (B) dispersal before selection (*move first*).

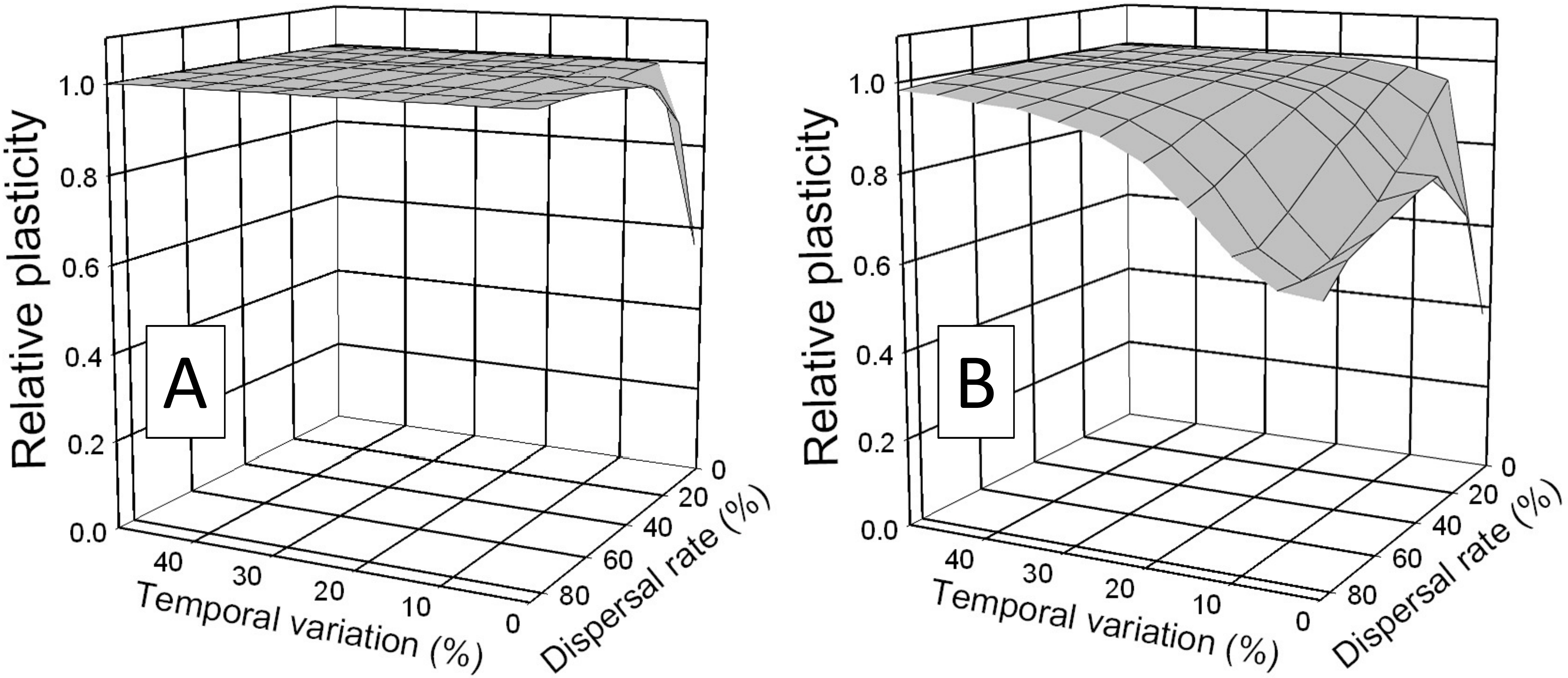

Figure S3. The interaction of dispersal rate and temporal variation of the local phenotypic optima on the evolution of phenotypic plasticity (mean P_{ij}) when selection occurs before dispersal (*select first*). Temporal variation is scaled as a percentage of the length of the environmental gradient. Dispersal was by the stepping stone migration pattern. (A) The environment changed once per generation after development but before selection and was independent among demes (pattern 1). (B) The environment changed before development and again before selection and was independent among demes (pattern 3).
